# Growth and regression of an intracranial vertebral artery dissecting aneurysm

**DOI:** 10.3389/fneur.2025.1566861

**Published:** 2025-05-07

**Authors:** Yalnaz Mohasin, Timo Krings

**Affiliations:** ^1^Royal College of Surgeons in Ireland, Al Muharraq, Bahrain; ^2^Division of Neurointerventional Radiology, Lahey Hospital & Medical Center-Beth Israel Lahey Health, UMass Chan Medical School, Boston, MA, United States; ^3^Department of Medical Imaging, University of Toronto, Toronto, ON, Canada; ^4^Singleton Department of Radiology, Texas Children’s Hospital and Baylor College of Medicine, Houston, TX, United States

**Keywords:** vertebral artery, arterial dissection, stroke, hemorrhage, spontaneous resolution

## Abstract

Intracranial vertebral artery dissecting aneurysms (VADAs) are rare vascular abnormalities with diverse presentations and unpredictable natural histories. Traditionally considered aggressive lesions with high mortality, emerging evidence has suggested some unruptured cases may undergo stabilization or even regression. This report details a 47-year-old patient presenting with ataxia and neck pain following a presumed traumatic dissection, leading to a diagnosis of a right vertebral artery dissection with mural hematoma formation. Serial imaging over two-years demonstrated progressive aneurysmal growth with mass effect at 6 weeks and 9 weeks, followed by stabilization at 12 months and subsequent complete regression of the aneurysm by 24 months. Conservative management was pursued due to patient preference, highlighting the importance of patient selection in decision making for VADAs. The observed spontaneous regression likely reflects a combination of mural hematoma reabsorption, closure of the dissecting flap, and robust collateral circulation. This case contributes to the evolving understanding of intracranial dissection aneurysms, emphasizing the potential for self-healing in select cases while reinforcing the need for individualized treatment strategies.

## Introduction

Intracranial vertebral artery dissecting aneurysms (VADAs) are rare yet clinically significant vascular abnormalities with diverse presentations and complex pathophysiological mechanisms. They often arise due to intramural hematoma formation within the arterial wall, stemming from either intimal tears or, less commonly, rupture of the vasa vasorum (1, 2). Unlike extracranial vertebral artery dissections, which typically have a more benign course and lower risk of spontaneous hemorrhage, intracranial VADAs are historically associated with higher rates of morbidity and mortality (3, 4).

Vertebral artery dissections can lead to variable clinical courses depending on the fate of the mural hematom, which may result in luminal narrowing, aneurysmal dilation causing mass effect, or subarachnoid hemorrhage (SAH) (5, 6). The higher risk of SAH in intracranial VADAs is linked to their thinner tunica media and adventitia, making them less stable than their extracranial counterparts (7). Historically, VADAs have been considered aggressive lesions with high rates of rupture and rebleeding, with mortality rates exceeding 40% in acute hemorrhagic presentations (8).

Although intracranial VADAs have often been managed with aggressive endovascular or surgical interventions, recent evidence suggests that a subset of these aneurysms may follow a more indolent course. Emerging literature has described cases where unruptured VADAs remain stable even regress spontaneously without intervention (9, 10). This contrasts traditional paradigm, that prioritize intervention due to the perceived high rupture risk of these aneurysms. The decision to intervene is often influenced by clinical presentation, aneurysm morphology, and interval changes on imaging (11–13). However, data from long-term follow-ups indicate that not all dissecting aneurysms require immediate treatment, particularly if they demonstrate stability on serial imaging (14).

Spontaneous regression of dissecting aneurysms is thought to occur through several mechanisms, including mural hematoma resorption, vessel wall remodeling, and thrombosis-induced occlusion of the dissecting segment (12, 13). Serial imaging has further demonstrated cases where conservative management was feasible, with aneurysms evolving into stable configurations, emphasizing need for individualized treatment strategies (9, 14). In particular, evidence suggests that extracranial vertebral artery dissections exhibit a higher rate of spontaneous resolution compared to intracranial lesions, which may be due to differences in vascular wall structure and biomechanical stress (4, 10).

The natural history of intracranial VADAs is highly variable, ranging from progressive enlargement to spontaneous resolution. Certain dissecting aneurysms initially present with significant mural hematoma growth yet resolve entirely under conservative management, as seen in this report. Such cases challenge the traditional assumption that all dissection aneurysms require intervention, particularly in asymptomatic or stable patients (2, 4).

While ruptured intracranial VADAs necessitate urgent intervention due to the high risks of rebleeding and mortality, the management of unruptured cases remains controversial (5, 8). This report describes a rare case of an intracranial VADA that demonstrated progressive growth followed by spontaneous regression, supporting the hypothesis that select cases may follow a more benign trajectory than previously believed. By integrating clinical findings with prior research, this report aims to refine our understanding of the natural history of VADAs and highlight the factors that may favor spontaneous healing (15, 16).

## Case discussion

A 47-year-old previously healthy patient with no prior history of stroke, aneurysm, or other cerebrovascular disease presented to the hospital with a two-day history of progressive ataxia. The patient described a sudden onset of right-sided neck pain radiating to the suboccipital region that began immediately following a vigorous golf swing. This pain was initially sharp and localized but later evolved into a persistent ache, prompting the patient to seek medical evaluation. The clinical presentation, coupled with the patient’s relatively young age and absence of traditional stroke risk factors such as hypertension, diabetes, smoking, or hyperlipidemia, raised suspicion for a nonatherosclerotic cause of ischemia.

**Figure 1:**
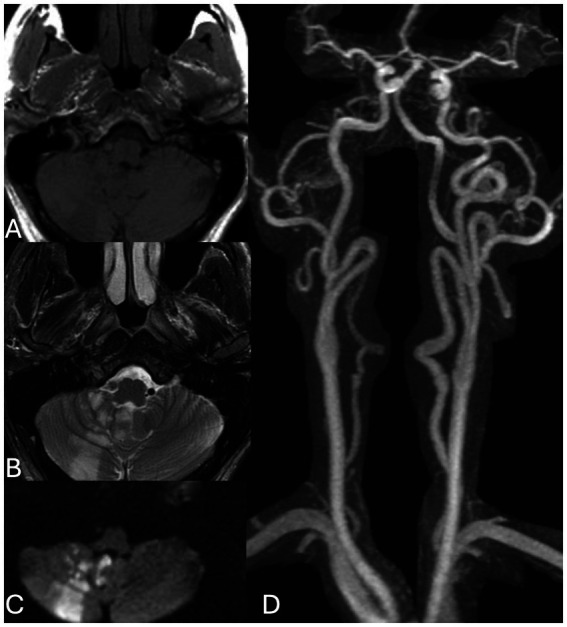
Baseline MR imaging during acute presentation: axial T1 **(A)**, T2 **(B)**, and DWI **(C)** sequences as well as a contrast enhanced MRA **(D)** demonstrate a missing flow void in the right vertebral artery, acute ischemia in the PICA territory and no contrast in the right V4 segment in keeping with an acute vertebral artery dissection. There is no aneurysmal dilatation at this point. Note multiple irregularities of the bilateral V2 segments as well as elongated arteriopathy in keeping with potential underlying collagenopathy.

Neuroimaging revealed acute ischemia within the right posterior inferior cerebellar artery (PICA) territory, confirmed on diffusion-weighted imaging (DWI) (Figure 1). Further evaluation with axial T1-weighted magnetic resonance imaging (MRI) demonstrated a mural hematoma in the right vertebral artery, a hallmark of acute arterial dissection. Additionally, the absence of flow voids in the right PICA region suggested vascular compromise or occlusion. Magnetic resonance angiography (MRA) of the head and neck confirmed the presence of an eccentric thrombus with vessel wall irregularity and arterial narrowing proximal to the V4 segment, consistent with an intracranial vertebral artery dissection (VAD). High-resolution MRI has been shown to reveal evolving mural hematomas in VADs even before classic angiographic signs emerge (17). This is exemplified in the early phase MRI and DWI findings, which show acute ischemia in the PICA territory and intramural hematoma within the right vertebral artery (Figure 1). The dissection extended into the Atlanta loop, a common site for mechanical stress-related injury due to the artery’s tortuous course around the atlas vertebra.

Vessel wall imaging revealed no evidence of fibromuscular dysplasia, vasculitis, or underlying vascular abnormalities, ruling out inflammatory or congenital vessel wall disease as contributing factors. Given the absence of significant comorbidities or systemic conditions that could predispose the patient to stroke, the clinical history of abrupt neck motion provided a plausible explanation for the arterial injury. The patient was initiated on anticoagulation therapy to prevent further thromboembolic events.

### Follow-up and imaging progression

**Figure 2:**
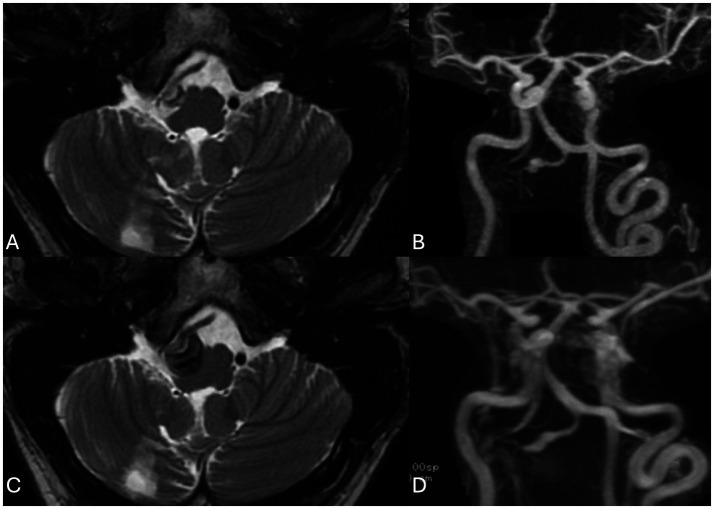
MR at 6 weeks (**A**: axial T2 weighted scans), (**B**: contrast enhanced MRA) and 9 weeks (**C**: axial T2 weighted scans), (**D**: contrast enhanced MRA) following the acute presentation. After 6 weeks there is no an aneurysmal appearance of the right V4 segment with crescent shaped thrombus of varying ages as well as retrograde filling of the V4 segment with luminal dilatation. At 9 weeks there is stability of the luminal dilatation on MRA but continued growth and new mass effect of the mural hematoma that has developed an onion skin layering with new mass effect upon the medulla oblongata.

At 6 weeks, follow-up MRI and MRA demonstrated fusiform dilatation of the vertebral artery and eccentric luminal thickening, indicating early aneurysmal formation (Figure 2).

At 9 weeks, subsequent imaging revealed progression of the mural hematoma, while the luminal dilatation remained unchanged (Figure 2). Endovascular intervention with parent vessel occlusion was offered to the patient; however, he declined and opted for further follow-up imaging as he remained asymptomatic.

**Figure 3:**
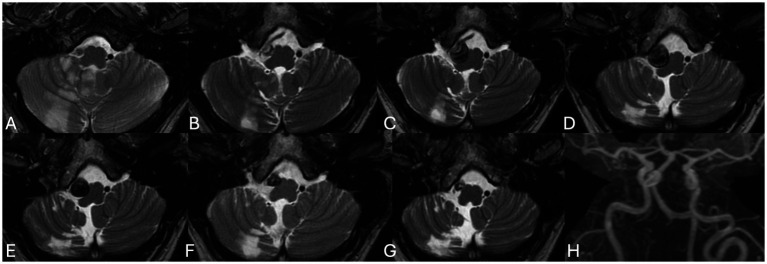
Axial T2 weighted scans at presentation **(A)**, six weeks **(B)**, nine weeks **(C)**, twelve weeks **(D)**, six months **(E)**, one year **(F)** and two years **(G)** as well as contrast enhanced MRA at 2 years **(H)** demonstrate the evolution of this patient’s dissection from initial growth to stabilization to subsequent regression of the aneurysm and its associated mass effect.

At 12 months, MRI showed continued abluminal growth with progressive mass effect due to expansion of the mural hematoma. Endovascular management was strongly recommended, but the patient again declined intervention (Figure 3).

At 18 and 24 months, follow-up imaging demonstrated stabilization of the dissection, with no evidence of new ischemic changes or worsening vascular compromise. By 24 months, complete regression of the mural hematoma and thrombus was observed, consistent with the natural healing process of the dissection. The timeline of imaging findings is summarized in Table 1.

**Table 1:** Timeline of imaging modality and key clinical findings at patient follow-up.

Follow-up interval	Imaging modality	Key findings
Initial presentation	CTA head & neck (Figure 1)	Intramural hematoma, right intracranial VADA
6 weeks	MRI & MRA	Progressive mural thickening, aneurysm growth
9 weeks	MRI & MRA (Figure 2)	Increased aneurysm size, mild brainstem compression
12 months	MRI & MRA	No further growth, stable configuration
18 months	MRI & MRA	Stabilization of mural hematoma
24 months	MRI & MRA (Figure 3)	Complete resolution of aneurysm

Clinically, the patient’s ataxia and neck pain completely resolved, and no new neurological deficits were observed. Given the favorable progression and imaging findings, continued conservative management was pursued. On final imaging, the vessel had nearly remodeled to its prior state. This case highlights the importance of considering intracranial vertebral artery dissections in younger patients presenting with posterior circulation ischemia, especially when precipitated by activities involving abrupt neck motion. It reveals the potential for spontaneous obliteration of an initially growing dissecting aneurysm and reinforces the need for individualized treatment decisions.

## Discussion

### Pathophysiology of intracranial vertebral artery dissection aneurysms

Intracranial vertebral artery dissecting aneurysms (VADAs) arise from an intimal tear or rupture of the vasa vasorum, leading to intramural hematoma formation and subsequent vessel wall remodeling (1, 2, 17). These aneurysms are often dynamic in nature, with the potential for either progressive expansion, spontaneous thrombosis, or in rare cases, complete resolution. Unlike extracranial vertebral artery dissections, which typically undergo spontaneous resolution through remodeling and recanalization, intracranial VADAs are associated with a higher risk of aneurysm formation and rupture due to the lack of an external elastic lamina (3, 4, 11).

Although most unruptured VADAs remain stable, a subset of cases exhibit progressive dilating requiring intervention (4). This case challenges this expectation, demonstrating that spontaneous healing through mural hematoma resorption and endothelial remodeling is possible under certain conditions. One proposed mechanism involves closure of the dissecting flap, limiting further accumulation of blood and promoting hematoma resorption (13, 18). This self-limiting behavior parallels healing observed in extracranial dissections, though intracranial lesions are generally less stable due to differences in vessel wall composition and hemodynamics (12).

In our case, the absence of significant hemodynamic stress or a chronic inflammatory environment likely facilitated spontaneous regression. Additionally, robust collateral circulation could have reduced vascular strain, reducing flow-related strain on the dissected segment and contributing to the observed regression (2, 10). Understanding the interplay of these factors could provide deeper insight into identifying which patients may be suitable for conservative management rather than immediate intervention.

### Comparative cases and spontaneous regression

This case presents a unique and rare natural history of an intracranial vertebral artery dissection (VADA) associated with a posterior inferior cerebellar artery (PICA) infarct. The patient initially experienced sudden-onset, sharp right-sided neck pain radiating to the suboccipital region, followed by progressive mural hematoma mimicking a partially thrombosed aneurysm. Over time, the aneurysmal dilation and mural hematoma growth mimicked a partially thrombosed aneurysm. Despite its concerning progression, the aneurysm underwent complete spontaneous regression and vascular remodeling without any endovascular intervention.

Such an outcome is exceedingly rare in intracranial VADAs, as most reports describe either stable aneurysm morphology or progressive worsening necessitating treatment. To our knowledge, this is the first report describing a growing dissection aneurysm that subsequently regressed completely. The natural history observed in this case challenges existing paradigms regarding the necessity of endovascular management in all growing aneurysmal lesions. A similar outcome was reported by Chida et al. (19), who described spontaneous regression of a VADA under conservative management. It highlights that conservative observation may be a viable strategy in select patients under close surveillance.

### Literature review

Spontaneous resolution of intracranial VADAs is extremely rare but has been documented in select cases where aneurysms remained unruptured and exhibited controlled remodeling. The majority of reported cases describe mural hematoma progression following dissection without subsequent resolution (2, 9, 10). A review of intraranial dissection cases found that, while extracranial dissections often heal without intervention, intracranial lesions are more likely to progress to rupture or thrombosis-induced occlusion rather than spontaneous regression (4, 8). Rare reports describe cases similar to ours such as Dmytriw et al. (6) reporting a small subset of dissecting aneurysms undergoing partial regression, but none exhibited complete resolution. While Matsumoto et al. (13) suggested that regression may be possible in aneurysms with low intramural thrombus burden, which aligns with our case findings. Maeoka et al. (20) also further reported spontaneous resolution of a dissecting aneurysm in a pediatric patient, further supporting this possibility.

Additionally, a large cohort study examining natural history outcomes of posterior circulation dissecting aneurysms demonstrated that the likelihood of spontaneous resolution was highest in younger patients with stable aneurysm morphology, lack of significant intramural thrombus burden, and absence of flow-related stressors (10, 18). The mechanisms behind spontaneous regression remain poorly understood, but our case offers insight into potential pathways contributing to self-limiting aneurysmal behavior.

### Potential mechanisms of spontaneous regression

Several mechanisms have been proposed to explain the spontaneous regression of dissecting aneurysms, including mural hematoma resorption, endothelial repair, vascular remodeling, and hemodynamic adaptation. Over time, intramural thrombus may degrade through fibrinolytic processes, leading to a gradual reduction in mural expansion (21). Secondly, additionally, neointimal formation through fibroblast and smooth muscle proliferation can reinforce vessel integrity, stabilizing the aneurysm wall (18). Histological studies have shown that neointimal formation and thrombus organization can stabilize the dissected segment (22). Vascular remodeling, including collateral vessel formation and redirection of blood flow, may reduce intramural pressure and facilitate vessel healing (16, 23). Hemodynamic adaptation, such as decreased flow velocity in the dissected segment, can promote thrombosis and subsequent remodeling, ultimately leading to resolution (13). In this case, the absence of rapid aneurysm expansion, significant intramural thrombus burden, or wall irregularity likely contributed to a more favorable natural history, allowing for vessel healing rather than rupture.

### Implications for management

The management of intracranial VADAs remains controversial, particularly in cases that remain unruptured and asymptomatic. The current standard of care favors early endovascular intervention in high-risk lesions, including those with aneurysmal dilation, worsening mass effect, or evidence of progressive thrombosis (5, 8). However, the growing body of evidence on conservative management suggests that a subset of patients may achieve favorable outcomes without invasive intervention. This finding is supported by Guan et al. (24), who reported similar clinical outcomes in both conservative and endovascularly managed unruptured VADAs. They found that patients with stable unruptured VADAs had comparable long-term outcomes regardless of whether they underwent conservative or endovascular management, suggesting the viability of non-interventional approaches in select cases. Treatment selection—whether deconstructive or reconstructive—should be informed by lesion morphology, flow dynamics, and anatomical location, particularly in vertebrobasilar dissections (25). Longitudinal studies indicate that stable, unruptured aneurysms with minimal mural hematoma growth over serial imaging may follow a benign natural course (4, 14).

This case reinforces the importance of risk stratification when considering treatment options for unruptured intracranial VADAs. Aggressive intervention is warranted in cases demonstrating rapid expansion, irregular morphology, or mass effect on adjacent structures. However, in patients exhibiting stable imaging findings, conservative management with serial follow-up may be a justifiable alternative (2, 10). Furst et al. (26) similarly reported favorable outcomes in patients with stable unruptured VADAs managed conservatively. Additionally, emerging research suggests that certain imaging biomarkers, such as vessel wall enhancement on high-resolution MRI and dynamic luminal changes, may predict aneurysm behavior over time (6). Further studies are needed to establish a risk stratification model that can guide decision-making in clinical practice.

## Conclusion and broader implications

This case provides further evidence that spontaneous regression of intracranial VADAs, although rare, is possible in select patients. It reinforces the need for careful patient selection when considering conservative management, serial neuroimaging to monitor aneurysm behavior over time, and continued research into predictive factors for spontaneous resolution. While endovascular intervention remains the gold standard for high-risk aneurysms, this case suggests that not all expanding intracranial dissecting aneurysms require immediate treatment. Future research should focus on identifying biomarkers and imaging criteria to reliably predict aneurysm behavior, potentially leading to more individualized treatment algorithms. By documenting a rare case of spontaneous resolution, this report highlights the potential for self-healing capacities in intracranial vascular pathologies and emphasizes the need for a nuanced approach to VADA management.
